# A Novel Supplement Attenuates Oxidative Stress-Induced TDP-43-Related Pathogenesis in TDP-43-Expressed Cells

**DOI:** 10.1155/2021/6773260

**Published:** 2021-09-27

**Authors:** Eun Jin Yang

**Affiliations:** KM Medicine Science Research Division, Korea Institute of Oriental Medicine, 1672 Yuseong-Daero, Yuseong-Gu, Daejeon 305811, Republic of Korea

## Abstract

Amyotrophic lateral sclerosis (ALS) is caused by selective the loss of spinal motor neurons by multifactorial pathological mechanisms and results in muscle atrophy. Incidence rates of ALS are increasing over time, but there are no effective treatments at present due to limitations on approved therapies (riluzole and edaravone). Therefore, this study investigated whether combined treatment with Bojungikgi-tang and riluzole could act synergistically in transactive response DNA-binding protein 43 (TDP-43) stress granule cells. To examine the effect of combined treatment on oxidative stress-induced cell death, the CCK8 assay was performed for the detection of cell viability. The expression of oxidative stress-induced proteins was determined by Western blot. Quantification of sodium arsenite-induced reactive oxygen species (ROS) was measured in TDP-43 stress granular cells using 2,7-diacetyl dichlorofluorescein diacetate. To investigate the effect of combined treatment on TDP-43 aggregation, immunofluorescence and immunoblotting were performed in TDP-43 stress granular cells. This combined treatment alleviated oxidative stress-induced cell death by increasing the expression levels of antioxidation proteins, such as heme oxygenase-1 and B cell lymphoma-2-associated X protein. Furthermore, it reduced oxidative stress-induced TDP-43 aggregates and lowered the levels of autophagy-related proteins, including p62, light chain 3b, and ATG8, in TDP-43-expressing cells. Our results suggest that this combined treatment could be helpful for autophagy regulation in other neurodegenerative diseases.

## 1. Introduction

Amyotrophic lateral sclerosis (ALS) is caused by selective loss of spinal motor neurons by multifactorial pathological mechanisms and results in muscle atrophy [1]. ALS-related genes include mutations in superoxide dismutase 1, fused in sarcoma (FUS), transactive response DNA-binding protein 43 (TDP-43), C9orf72, optineurin, sequestosome 1, ubiquilin 2, dynactin, MATR3, and valosin-containing protein (VCP) [2]. The pathological mechanisms of ALS include excitotoxicity, apoptosis, inflammation, and oxidative stress at the cellular level [2]. TDP-43 is involved in frontotemporal lobar degeneration (FTLD), and ALS and its pathological mechanisms include oxidative stress [3], neuroinflammation [4], and mitochondrial dysfunction [5]. In particular, oxidative stress activates pathological pathways leading to neuronal degeneration and motor neuron death [6]. Furthermore, TDP-43 has been associated with the RNA-related metabolism, stress granule formation, the ubiquitin-proteasome system (UPS), autophagy, and mitochondrial dysfunction [7]. The TDP-43 protein is a significant component of the characteristic ubiquitinated neuronal inclusions [8, 9]. Abnormal TDP-43 is displaced from the nucleus to the cytoplasm and forms aggregates due to defects in the machinery regulating protein homeostasis, such as the autophagy-lysosome pathway and UPS [10]. The research is inconsistent as to whether the aggregation of TDP-43 protein is beneficial or induces neurotoxicity. Xu et al. and Wegorzewska et al. have shown that mutant TDP-43 protein is toxic to neurons in several TDP-43 animal models [11, 12]. However, Vander Broeck et al. suggested that the loss of TDP-43 induces TDP-43-related proteinopathies rather than toxic effects by aggregates [13]. Significant research has focused on finding a drug to prevent or alleviate TDP-43-related disease conditions. The multiple complex pathomechanisms have complicated finding an effective drug for ALS. Although riluzole has been used to treat patients with ALS, it does not appear to extend patients' survival. Furthermore, riluzole does not improve neuropathology in TDP-43 expressed animal models [14, 15].

Herbal medicines, such as the Huolingshengji formula and Jiawei Sijunzi, have been shown to improve neurological and muscle function in patients with ALS [16, 17]. In patients with ALS, herbal medicines, such as the Huolingshengji formula and Jiawei Sijunzi, have been shown to improve neurological and muscle functions [16, 17]. Chang et al. showed that berberine could be a potential therapy as an activator of mTOR-autophagy signals TDP-43-related neuropathology [18]. Bojungikgi-tang (BJIGT) is a traditional herbal medicine in traditional Chinese medicine (TCM), is known as Bu Zhong Yi Qi Tang (BZYQT) in Chinese and Hochuekkito in Japanese, and comprises eight herbs. Several studies have demonstrated the effects on tumor suppression [19], chronic fatigue syndrome [20], and immune function [21]. Furthermore, we have shown that BJIGT treatment improved motor activity with anti-inflammatory and antioxidative effects in the spinal cord and gastrocnemius muscle of the hSOD1^G93A^ animal model [22]. We have investigated the effects of combined treatment with BJIGT and riluzole in TDP-43 stress granular cells to examine the synergistic effect of combined treatment. The combined treatment reduced oxidative stress-induced cell death and regulated autophagy dysfunction compared with sodium arsenite (SA) only-treated TDP-43 cells. From those findings, we suggest that combined treatment could be helpful for aggregate clearance in other neurodegenerative diseases.

## 2. Methods

### 2.1. Cell Subculture and Drug Preparation

TDP-43 stress granules cells were purchased from Innovative Technologies in Biological Systems (Innoprot, Spain). The TDP-43 stress granules cells were maintained in Dulbecco's MEM/Nut MIX F-12 medium (Sigma Aldrich, USA) supplemented with 10% fetal bovine serum (Thermo Fischer Scientific, CA, USA) and 5 *μ*g/mL puromycin (Thermo Fischer Scientific, CA, USA) and 80 *μ*g/mL hygromycin (Thermo Fischer Scientific, CA, USA). Cells were grown at 37°C and 5% CO_2_ incubator and subcultured every 3 days. For the induction of fluorescent TDP-43 expression, 50% confluence cells were added 5 mM isopropyl *β*-d-1-thiogalactopyranoside (IPTG) in a complete medium. For the study, BJIGT and riluzole were purchased from Hankookshinyak (Chungnam, Korea) and Millipore (Billerica, MA, USA). BJIGT was dissolved with distilled PBS (dPBS) and filtered with 0.45 *μ*m syringe filter. Riluzole was dissolved in dimethyl sulfoxide (Sigma Aldrich, USA).

### 2.2. Cell Viability (CCK-8 Assay)

Cell viability was observed using the Cell Counting Kit-8 (CCK-8) (Sigma Aldrich, USA). TDP-43 stress granules cells were subcultured in a 96-well plate, pretreated with 100 *μ*g/mL BJIGT or 50 *μ*M riluzole or combined BJIGT (100 *μ*g/mL) and riluzole (50 *μ*M), and then exposed to 150 *μ*M sodium arsenite (SA) for 18 h or sterilized water as a solvent control. Treated cells were added 10 *μ*L of CCK-8 solution. The assay was repeated three times. The optical density value was measured at absorbance 450 nm by a microplate reader (SpectraMAX 340, molecular devices, USA) for cell viability.

### 2.3. Western Blot

After incubation with 150 *μ*M SA for 18 h, the cells were rinsed twice with PBS. We used radioimmunoprecipitation assay (RIPA) buffer (50 mM Tris-HCl, pH 8.0, 150 mM NaCl, 1% NP-40, 0.5% sodium deoxycholate, and 0.1% sodium dodecyl sulfate (SDS)), which included phosphatase and protease inhibitors (GenDEPOT, Barker, TX, USA), to lyse the cells; this sample was called “RIPA-soluble.” The lysed cells were centrifuged at 4°C at 12,000 rpm for 20 min. We evaluated the supernatants with the bicinchoninic acid (BCA) protein assay (Thermo Fischer Scientific, CA, USA) to determine the protein concentration of the lysates. The RIPA-insoluble pellets were lysed with 10% SDS buffer, sonicated, and supplemented with SDS-polyacrylamide gel electrophoresis (PAGE). The soluble and insoluble proteins were separated on 4–12% SD-PAGE gels and transferred to PVDF membranes. PVDF blocked with 5% skim milk for elimination of nonspecific binding and then incubated with the following primary antibodies: heme oxygenase (HO) 1, ATG7, and tubulin (Abcam, Cambridge, MA, USA); B cell lymphoma-2-associated X protein (Bax) and green fluorescent protein (GFP) (Santa Cruz Biotechnology, Santa Cruz, CA, USA); microtubule-associated protein 1A/1B light chain (LC) 3b and P62 (Cell Signaling Technology, Danvers, MA, USA); and ubiquitin (Vector Laboratories, Burlingame, CA, USA) at 4°C overnight. After rinsing with 0.1% Tween in Tris-buffered saline, the PVDF was incubated with anti-rabbit of anti-mouse IgG antibodies (Santa Cruz Biotechnology). For chemiluminescent detection, the membrane was incubated with SuperSignal West Femto Substrate Maximum Sensitivity Substrate (Thermo Fisher Scientific, CA, USA). Immunoblots were detected using a ChemiDoc Imaging System (BioRad), and quantification was performed using ImageJ software (National Institutes of Health, Bethesda, MD, USA).

### 2.4. Determination of Reactive Oxygen Species Generation

Quantification of SA-induced reactive oxygen species (ROS) was measured in TDP-43 stress granular cells using 2,7-diacetyl dichlorofluorescein diacetate (DCFH-DA). DCFH-DA penetrates the intracellular matrix of cells and oxidizes DCFH-DA, depending on ROS, and then forms fluorescent DCF. TDP-43 stress granular cells (4 × 10^3^/mL) were treated with DCFH-DA (5 *μ*M) and incubated at 37°C for 10 min. Stained cells were rinsed with dPBS and added trypsin to remove excess DCFH-DA. Fluorescent intensity was performed with excitation, and emission filters were set at 490 nm and 535 nm, respectively, using SpectraMax i3 (Molecular devices, San Jose, USA).

### 2.5. Immunofluorescence and Aggregation Analysis

TDP-43 stress granular cells were treated with 70% ice-cold ethanol on ice for fixation. Permeabilized cells with PBST (PBS, 0.5% Triton X-100) were blocked with 5% bovine serum albumin. After blocking, TDP-43 stress granular cells were incubated with anti-GFP, at 4°C overnight. TDP-43 stress granular cells were treated with Alexa Fluor 488-conjugated goat anti-mouse immunoglobulin G secondary antibody (1 : 500; Invitrogen). The cell images were observed on an Eclipse Ti-U (Nikon, Tokyo, Japan) fluorescence microscope. For quantification of aggregates in cells, three representative microscopy fields per sample were analyzed. The aggregated cells by SA treatment were calculated by the number of GFP-positive aggregated cells/total number of cells.

### 2.6. Statistical Analyses

Statistical analysis was analyzed using GraphPad Prism 9.0 (GraphPad Software, San Diego, CA, USA), and barplot presented with the mean ± standard error of the mean (SEM) were indicated. Statistical significance of Western blotting, CCK8 assay, and ROS assay were analyzed using one-way analysis of variance followed by the Newman–Keuls test.

## 3. Results

### 3.1. Combined Herbal Medicine and a Drug Prevented SA-Induced Cell Death of TDP-43 Stress Granular Cells

**Figure 1:**
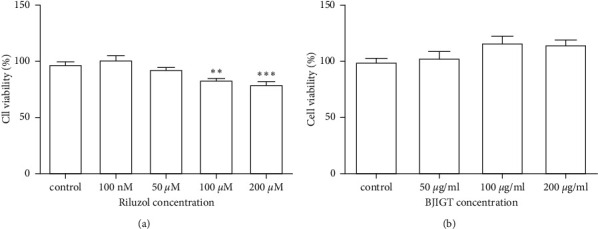
Cell viability under differing BJIGT and riluzole concentrations. TDP-43 stress granular cells incubated with riluzole (a) and BJIGT (b) at indicated concentrations for 24 h. Barplots are represented as a percentage of the control value. BJIGT, Bojungikgi-tang.

**Figure 2:**
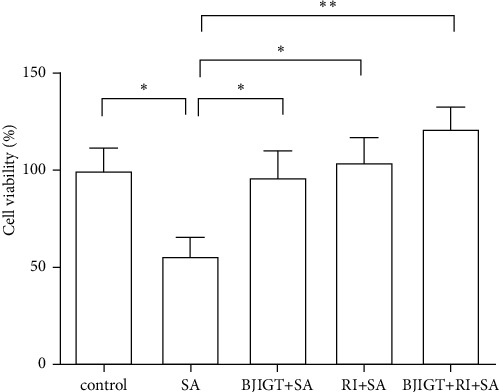
SA-induced cell death from BJIGT combined with riluzole in TDP-43-expressed stress granular cells. TDP-43 stress granular cells were pretreated with BJIGT (100 *μ*g/mL), riluzole (50 *μ*M), and combined BJIGT (100 *μ*g/mL)/riluzole (50 *μ*M) for 6 h before treatment of SA (150 *μ*M) for 18 h. Combined treatment prevented SA-induced cell death in TDP-43 stress granular cells. SA, sodium arsenite; BJIGT, Bojungikgi-tang; RI, riluzole.

We examined cell viability as a function of the concentration of riluzole and BJIGT in TDP-43 stress granular cells to determine cell toxicity. We observed a 14% reduction of cell viability with 100 *μ*M riluzole comparing with the control group (*p* < 0.01 and *p* < 0.001, Figures 1(a) and 1(b)). BJIGT did not reduce cell viability for doses up to 200 *μ*g/mL for 24 h in TDP-43 stress granular cells (Figures 1(a) and 1(b)). To investigate the combined treatment effect on SA-induced cell death, we preincubated cells with BJIGT (100 *μ*g/mL), riluzole (50 *μ*M), and combined BJIGT (100 *μ*g/mL) and riluzole (50 *μ*M) for 6 h before treatment with SA (150 *μ*M) over 18 h. BJIGT and riluzole treatment increased cell viability by 1.7- and 1.8-fold, respectively, compared with SA-treated TDP-43 stress granular cells. The combined treatment inhibited SA-induced cell death by 2.1-fold compared with the SA-treated group (*p* < 0.05 and *p* < 0.01, Figure 2). Our results suggest that combined treatment could have synergistic effects in the regulation of ALS pathology.

### 3.2. Combined Treatment Regulates Oxidative Stress-Induced Mechanism in TDP-43 Stress Granular Cells

**Figure 3:**
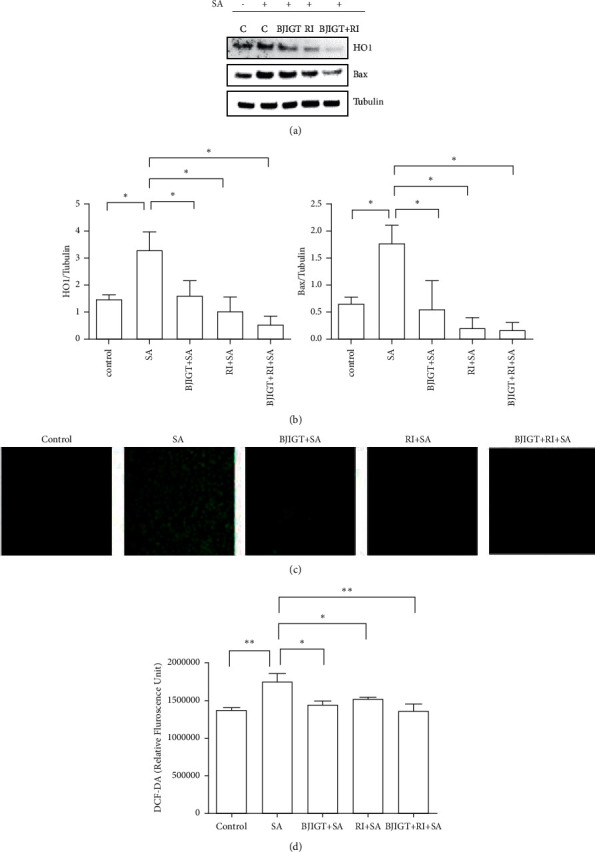
Oxidative stress effects of BJIGT combined with riluzole in TDP-43-expressed stress granular cells. TDP-43 stress granular cells were pretreated with BJIGT (100 *μ*g/mL), riluzole (50 *μ*M), and combined BJIGT (100 *μ*g/mL)/riluzole (50 *μ*M) for 6 h before treatment of SA (150 *μ*M) for 18 h. (a) Representative data of HO1 and Bax expression in TDP-43 stress granular cells. SA-induced oxidative stress-related proteins (HO1 and Bax) were reduced by BJIGT/riluzole in TDP-43-expressed stress granular cells. Tubulin was used as loading control. (b) Quantification of immunoblots. Bars represent the means ± SEMs of more than three independent experiments. ^*∗*^*P* < 0.05 vs. the SA-treated control group. (c) TDP-43 stress granular cells were treated with SA (150 *μ*M) for 18 h in the absence or presence of BJIGT (100 *μ*g/mL), riluzole (50 *μ*M), and combined BJIGT (100 *μ*g/mL)/riluzole (50 *μ*M), and then incubated with 5 *μ*M DCFH-DA. Cells were captured with a fluorescent microscope (40× magnification). (d) Relative fluorescence was quantified with a microplate reader. Bars represent the means ± SEMs of more than three independent experiments. ^*∗*^*P* < 0.05 and  ^*∗∗*^*p* < 0.01 vs. the SA-treated group. C, control; SA, sodium arsenite; BJIGT, Bojungikgi-tang; RI, riluzole.

To find the molecular mechanism of the combined treatment, we investigated the effects of oxidative stress-related proteins in SA-treated TDP-43 stress granular cells. As shown in Figures 3(a) and 3(b), we observed the reduction in HO1 and Bax protein levels by 2- and 3.2-folds, respectively, in BJIGT pretreatment in SA-treated TDP-43 expressing cells (*p* < 0.05). In addition, riluzole pretreatment reduced the expression of HO1 and Bax proteins by 3.1- and 8.7-folds, respectively, compared with SA-treated TDP-43 stress granular cells. Furthermore, the combined treatment significantly decreased the levels of HO1 and Bax proteins by 6- and 10.9-folds, respectively, compared with SA-treated cells (*p* < 0.05). To confirm the antioxidation effect of the combined treatment, we performed a DCFDA assay in SA-treated TDP-43 cells in the presence or absence of BJIGT and riluzole. The level of ROS generation using DCF fluorescence intensity increased by 1.2-fold in SA-treated cells compared with the generation in control (*p* < 0.05 and *p* < 0.01, Figures 3(c) and 3(d)). Interestingly, BJIGT and riluzole treatment reduced ROS production by 1.2- and 1.1-folds, respectively, compared with the SA-treated group. The combined treatment reduced 1.3-fold fluorescence intensity induced by SA treatment in TDP-43 stress granular cells (*p* < 0.01, Figures 3(c) and 3(d)).

### 3.3. Combined Treatment Regulated SA-Induced Autophagy Dysfunction in TDP-43 Stress Granular Cells

Ubiquitinated or hyperphosphorylated TDP-43 is detected in the nucleus due to oxidative stress or inflammation. It interacts with autophagy and UPS-related ubiquitin [9, 23].

**Figure 4:**
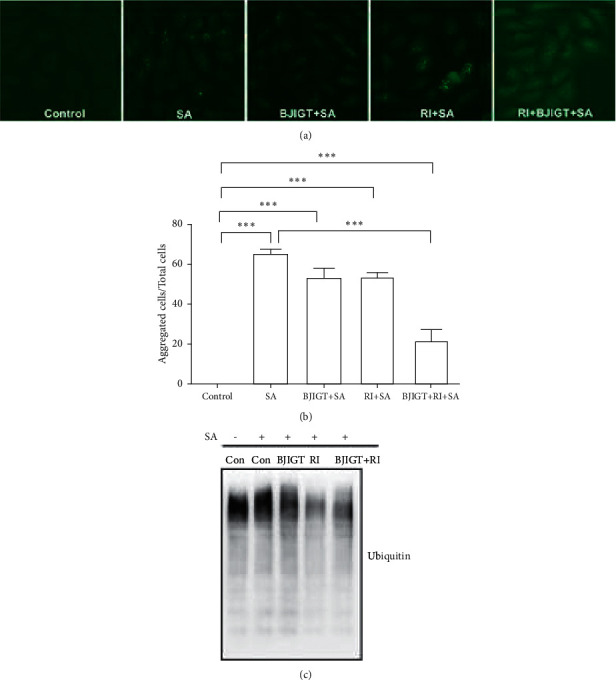
Aggregation effects of BJIGT combined with riluzole in TDP-43-expressed stress granular cells. TDP-43 stress granular cells were pretreated with BJIGT (100 *μ*g/mL), riluzole (50 *μ*M), and combined BJIGT (100 *μ*g/mL) and riluzole (50 *μ*M) for 6 h before treatment with SA (150 *μ*M) for 18 h. (a) The cells treated with anti-GFP immunofluorescence, the primary antibody, and captured with a fluorescent microscope (200× magnification). (b) Quantification of inclusions. Three representative microscopy fields per sample were analyzed. The aggregated cells by SA treatment were calculated by the number of GFP-positive aggregated cells/total number of cells. (c) Cell lysates identified with immunoblots using antiubiquitin. Con, control; SA, sodium arsenite; BJIGT, Bojungikgi-tang; RI, riluzole.

To investigate the effect of combined treatment on TDP-43 aggregation, we performed immunofluorescence and immunoblotting in TDP-43 stress granular cells. As shown in Figure 4, we found TDP-43 aggregates in SA-treated TDP-43-expressed cells. The combined treatment significantly reduced the aggregates by 2.9-fold in SA-treated TDP-43 expressed cells (*p* < 0.001, Figures 4(a) and 4(b)). Furthermore, we found that combined treatment reduced ubiquitinated high molecular proteins in SA-treated TDP-43 expressing cells compared with SA-treated TDP-43 expressing cells (*p* < 0.001, Figure 4(c)). These findings suggest that combined treatment can regulate SA-induced autophagy dysfunction in TDP-43-expressed cells.

**Figure 5:**
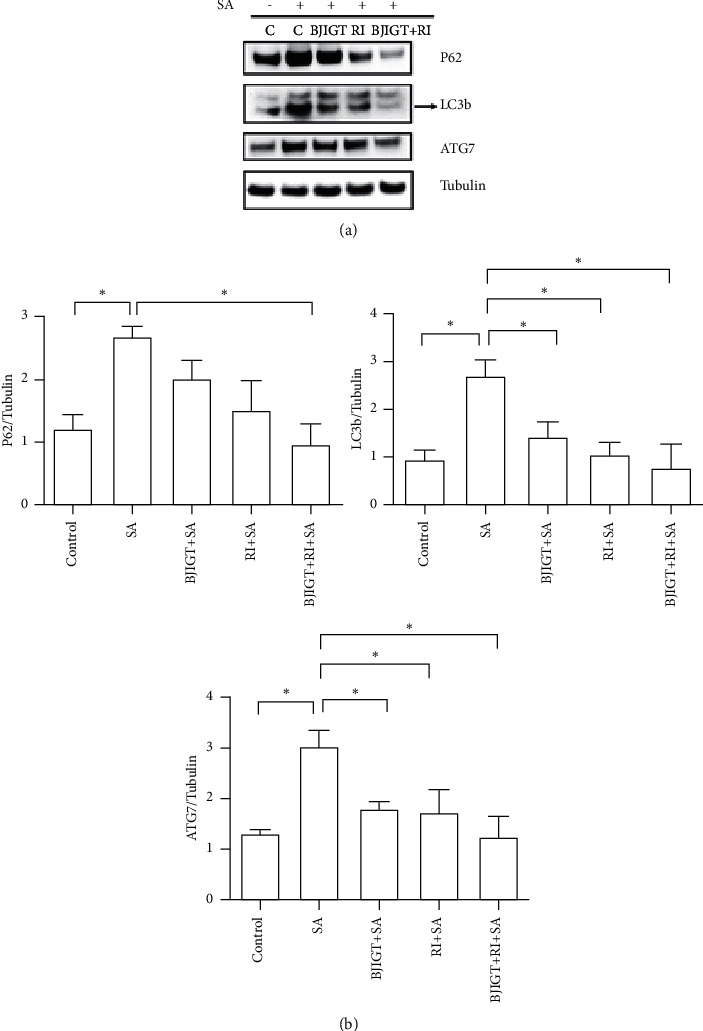
Autophagy function effects of BJIGT combined with riluzole in TDP-43-expressed stress granular cells. Cell lysates were used to detect P62, LC3b, and ATG7 expression by Western blots. Combined treatment reduced SA-induced autophagy dysfunction in TDP-43 stress granular cells. (a) Representative data of autophagy-related protein expressions (P62, LC3b, and ATG7) in TDP-43 stress granular cells. Tubulin was used as loading control. (b) Quantification of immunoblots. Bars show the means ± SEMs of more than three independent experiments. ^*∗*^*P* < 0.05 vs. the SA-treated control group.

To demonstrate the combined treatment effect on autophagy function, we investigated the levels of autophagy-related proteins, including P62, LC3b, and ATG7, in TDP-43-expressing cells. SA treatment significantly increased the expression levels of P62, LC3b, and ATG7 by 2.2-, 2.7-, and 2.3-folds, respectively, compared with the control in TDP-43 stress granular cells (*p* < 0.05, Figures 5(a) and 5(b)). However, combined treatment reduced P62, LC3b, and ATG7 protein levels by 2.8-, 3.4-, and 2.4-folds, respectively, compared with SA-treated TDP-43 stress granular cells (*p* < 0.05, Figures 5(a) and 5(b)).

## 4. Discussion

TDP-43 is important for regulating RNA biogenesis, such as splicing, translation, and stability [7]. However, TDP-43 overexpression and displacement to the cytoplasm are related to neurodegeneration, including motor nerve degeneration, as observed in ALS. Furthermore, TDP-43 proteinopathy induces neurodegenerative pathomechanisms, such as autophagy dysfunction [10], neuroinflammation [4], and mitochondrial dysfunction [5]. Several researchers have tried unsuccessfully to find an effective treatment or therapy for TDP-43-induced ALS. Although riluzole has been treated for patients with ALS, it is limited by improving survival for only 2-3 months [24], an unclear mechanism of action and adverse effects, such as nausea, asthenia, and gastrointestinal problems [25].

TCM has been used for disease treatment for centuries in China, Japan, and South Korea. BJIGT is a traditional herbal formula of TCM that has been known as BZYQT in Chinese and Hochuekkito in Japanese and comprises eight herbs. Several studies have demonstrated the effects on tumors [19], chronic fatigue syndrome [20], and immune function [21]. In our previous study, we demonstrated the anti-inflammatory and antioxidative effects of BJIGT in the muscle of the spinal cord of hSOD1^G93A^ mice [22]. In addition, Yu et al. showed that cotreatment with BZYQT and cisplatin enhanced apoptosis and autophagy, leading to increased intracellular ROS levels in human lung carcinoma A549/DDP cells (cisplatin-resistant cells) [26]. They suggested that BZYQT could be a potential chemotherapy sensitizer to reverse cisplatin resistance.

Oxidative stress is induced by excessive production of ROS by disruption of the antioxidant systems, leading to protein misfolding and causing inflammation and mitochondrial dysfunction [8]. In ALS, oxidative stress is a critical factor driving disease progression and disease pathogenesis [27]. TDP-43 is expressed as stress granules under cellular oxidative stress [28]. Liu et al. demonstrated that antioxidant oxidation resistance 1 extended survival and disease progression in an SOD1-mediated ALS animal model [29]. Edaravone was shown to remove lipid peroxide and hydroxyl radicals for neuronal protection and nitrosative stress in ALS [30]. However, acetylcysteine and creatine, as antioxidants, did not affect ALS symptom recovery [31]. This finding implies that oxidative stress is related to other pathological mechanisms, such as mitochondrial dysfunction, protein aggregation, and cytoskeletal dysfunction in ALS. Therefore, multiple target treatments are needed to improve complex diseases, such as ALS.

In this study, we investigated the effects of combined treatment with BJIGT and riluzole on SA-induced oxidative stress in TDP-43 stress granular cells. We found that combined treatment significantly reduced the expression levels of oxidative stress-induced HO1 and Bax proteins, as well as ROS generation. These findings suggest that cotreatment with BJIGT and riluzole could help the regulation of mitochondrial dysfunction in ALS. Zuo et al. demonstrated that TDP-43 aggregation induced by oxidative stress leads to mitochondrial imbalance [32]. SOD1-, TDP-43-, FUS-, and C9orf72-associated ALS causes toxicity by ubiquitin inclusions and is a common pathological feature of familial ALS or sporadic ALS [33, 34].

TDP-43 is expressed in the nucleus but represents cytosolic mislocalisation in a ubiquitinated or hyperphosphorylated form by oxidative stress or inflammation and interacts with autophagy and UPS-related ubiquitin [9, 23, 35]. Usually, autophagy and the UPS are important cellular mechanisms to clear protein aggregation. However, these functions are disrupted by oxidative stress in AD, PD, and ALS [36]. ALS genes are related to autophagic systems, such as p62, optineurin, VCP, ubiquilin 2, and TANK-binding kinase 1 (TBK1) [37]. Tanji et al. demonstrated that the interaction between TDP-43 and p62 was disrupted in the cerebral cortex of patients with FTLD-TDP [38].

As autophagy dysfunction is a critical pathological feature in ALS, AD, PD, and HD, Cipolat Mis et al. have investigated autophagy-regulating molecules in ALS models [39]. The autophagy inducer rapamycin is mTOR-dependently reduced protein aggregation, although it is only effective at the early stage of SOD1^G93A^ mice [40]. However, mTOR inhibition by rapamycin worsens the disease, although phosphatidic acid (an mTOR agonist) improved movement and developmental viability in a TDP-43-depleted *Drosophila* model [41]. Trehalose reduced SOD1 aggregation to prevent neuronal loss in SOD1^G93A^ mice [42] and induced TDP-43 clearance by autophagic activation in TDP-43 expressing SH-SY5Y cells [43]. These autophagic inductors, including rapamycin and trehalose, showed positive or negative effects in a nonclinical ALS model. Thus, further studies are needed in various experimental models before clinical translation. Our study found that combined treatment reduced insoluble ubiquitinated proteins, and TDP-43 aggregates via modulation of autophagy dysfunction under oxidative stress. These findings suggest that combined treatment could induce TDP-43 clearance via the autophagic degradation pathway. However, it is necessary to investigate autophagy regulation by combined treatment in other ALS-related animal models, such as hSOD1^G93A^ and FUS. Further studies will be performed to determine whether combined treatment activates mTOR-dependent or independent pathways in TDP-43-expressing cells. Furthermore, the interaction between BJIGT and riluzole should be investigated for translation into clinical studies.

## 5. Conclusion

In conclusion, this is the first study demonstrating that pretreatment with BJIGT and riluzole alleviates SA-induced oxidative stress and regulates autophagy in TDP-43 stress granule cells.

## Data Availability

The datasets that support the findings of this study are included within this article.
